# Effects of fatty acid activation on photosynthetic production of fatty acid-based biofuels in *Synechocystis *sp. PCC6803

**DOI:** 10.1186/1754-6834-5-17

**Published:** 2012-03-21

**Authors:** Qianqian Gao, Weihua Wang, Hui Zhao, Xuefeng Lu

**Affiliations:** 1 Key Laboratory of Biofuels, Qingdao Institute of Bioenergy and Bioprocess Technology, Chinese Academy of Sciences, No. 189 Songling Road, Qingdao 266101, China; 2 Graduate School of Chinese Academy of Sciences, Beijing 100049, China

**Keywords:** Biofuel, Fatty alcohol, Fatty alkane, Cyanobacteria, *Synechocystis *sp. PCC6803, Fatty acid activation

## Abstract

**Background:**

Direct conversion of solar energy and carbon dioxide to drop in fuel molecules in a single biological system can be achieved from fatty acid-based biofuels such as fatty alcohols and alkanes. These molecules have similar properties to fossil fuels but can be produced by photosynthetic cyanobacteria.

**Results:**

*Synechocystis *sp. PCC6803 mutant strains containing either overexpression or deletion of the *slr1609 *gene, which encodes an acyl-ACP synthetase (AAS), have been constructed. The complete segregation and deletion in all mutant strains was confirmed by PCR analysis. Blocking fatty acid activation by deleting *slr1609 *gene in wild-type *Synechocystis *sp. PCC6803 led to a doubling of the amount of free fatty acids and a decrease of alkane production by up to 90 percent. Overexpression of *slr1609 *gene in the wild-type *Synechocystis *sp. PCC6803 had no effect on the production of either free fatty acids or alkanes. Overexpression or deletion of *slr1609 *gene in the *Synechocystis *sp. PCC6803 mutant strain with the capability of making fatty alcohols by genetically introducing fatty acyl-CoA reductase respectively enhanced or reduced fatty alcohol production by 60 percent.

**Conclusions:**

Fatty acid activation functionalized by the *slr1609 *gene is metabolically crucial for biosynthesis of fatty acid derivatives in *Synechocystis *sp. PCC6803. It is necessary but not sufficient for efficient production of alkanes. Fatty alcohol production can be significantly improved by the overexpression of *slr1609 *gene.

## Background

Biofuel production from renewable sources is considered as a feasible solution to the energy and environmental problems we are facing. It is very important to explore and develop advanced biofuels alongside traditional biofuels such as bioethanol and biodiesel to ensure sufficient supply of renewable energy at a time when demand for energy is set to increase over the coming decades. Advanced biofuels possess higher energy density, hydrophobic properties and compatibility with existing liquid fuel infrastructure including fuel engines, refinery equipment and transportation/distribution pipelines, whilst serving as better alternatives to fuels produced from fossil fuels [1].

In terms of fuel properties the best replacement of petroleum fuels is "Petroleum Fuels". This means ideal biofuels produced from biological systems should be chemically similar to petroleum, such as fatty acid-based molecules including fatty alcohols and fatty alkanes [2].

As a candidate for biofuel-producing microbial systems, cyanobacteria have become more and more attractive due to their specific characteristics as photosynthetic bacteria.

Compared to generally utilized biofuel-producing microbes such as *E. coli *and *S.cerevisiae*, cyanobacteria are photosynthetic microbes, which can convert solar energy and carbon dioxide more efficiently into biofuels in one biological system. In contrast to plants and eukaryotic algae, cyanobacteria are prokaryotic microbes with the ability to grow a lot faster. Genetic engineering platforms for cyanobacteria are well established and they are highly tolerable to heterogeneous genes. So far over 40 genomic sequences of cyanobacteria strains are available, therefore genetic information on cyanobacteria are relatively robust http://genome.kazusa.or.jp/cyanobase. This makes genetic engineering toward efficiently producing biofuels in cyanobacteria to be a more realistic and feasible option [3-5].

**Figure 1:**
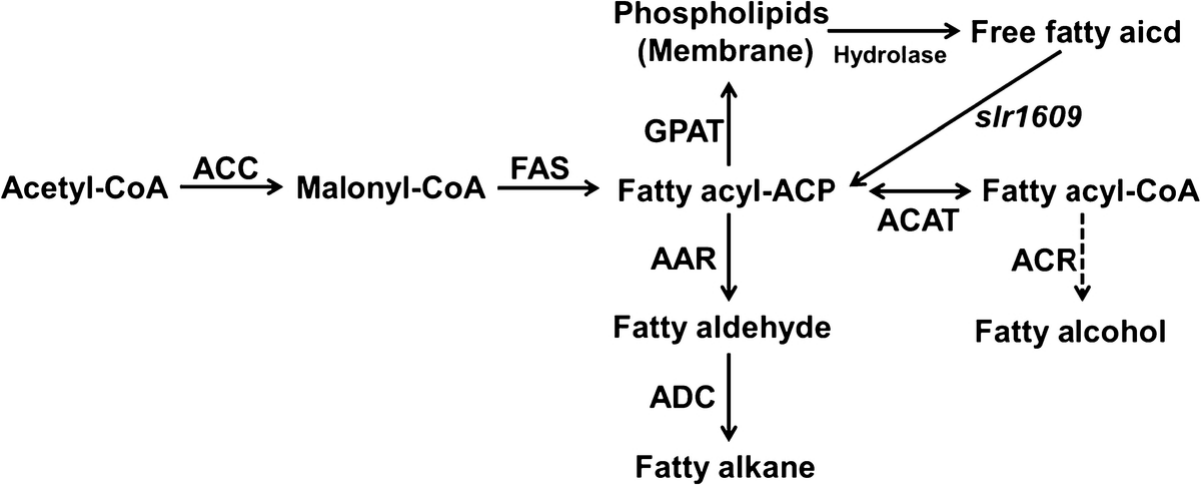
**The overall pathway of the fatty acid, fatty alcohol and fatty alkane in *Synechocystis *sp. PCC 6803**. Dash arrow represents non-native and heterologously introduced pathway. ACP, acyl carrier protein; AAR, acyl-ACP reductase; ADC, aldehyde decarbonylase; ACC, acetyl-CoA carboxylase; FAS, fatty acid synthase; GPAT, glycerol-3-phosphate-acyl transferase; ACAT, acyl-CoA-ACP transferase; ACR, acyl-CoA reductase.

Recently, the alkane biosynthetic pathway was identified in cyanobacteria with two enzyme families including an acyl carrier protein (ACP) reductase (AAR) and an aldehyde decarbonylase (ADC) [6]. Genes associated with an alcohol-forming fatty acyl-CoA reductase (FAR) have not been reported in cyanobacteria, C16:0 and C18:0 alcohols can be produced by engineered cyanobacteria containing the FAR gene derived from jojoba [7] or *Arabidopsis thaliana *[4]. The overall pathway of the fatty acid, fatty alcohol and fatty alkane in wild-type or engineered *Synechocystis *strains were illustrated in Figure 1.

The fatty acid molecules must be activated to fatty acyl-thioesters by fatty acyl-CoA synthetase (ACS, EC 6.2.1.3) or fatty acyl-ACP synthetase (AAS, EC 6.2.1.20) prior to the synthesis of fatty alcohols and alkanes. Based on sequence identity analysis, *Synechocystis *sp. PCC 6803 encodes only a single candidate gene for fatty acid activation, annotated as AAS and designated as *slr1609 *[8]. The *slr1609*-deletion cyanobacteria mutant was incapable of utilizing exogenous fatty acids and thus secreted endogenous fatty acids into the medium. The detected free fatty acids are released from membrane lipids. The data suggest a remarkable turnover of lipids and a role of AAS activity in recycling the released fatty acids [8].

The overall pathway of the fatty acid, fatty alcohol and alkane in wild-type or engineered *Synechocystis *strains are illustrated in Figure 1. *Synechocystis *sp. PCC6803 mutant strains with either overexpression or deletion of *slr1609 *gene have been constructed in this study. The results indicated that the AAS gene was metabolically crucial for production of free fatty acids and fatty acid derivatives in *Synechocystis *sp. PCC6803.

## Results and discussion

Construction of *Synechocystis *sp. PCC6803 mutants with either overexpression or deletion of *slr1609 *gene

**Figure 2:**
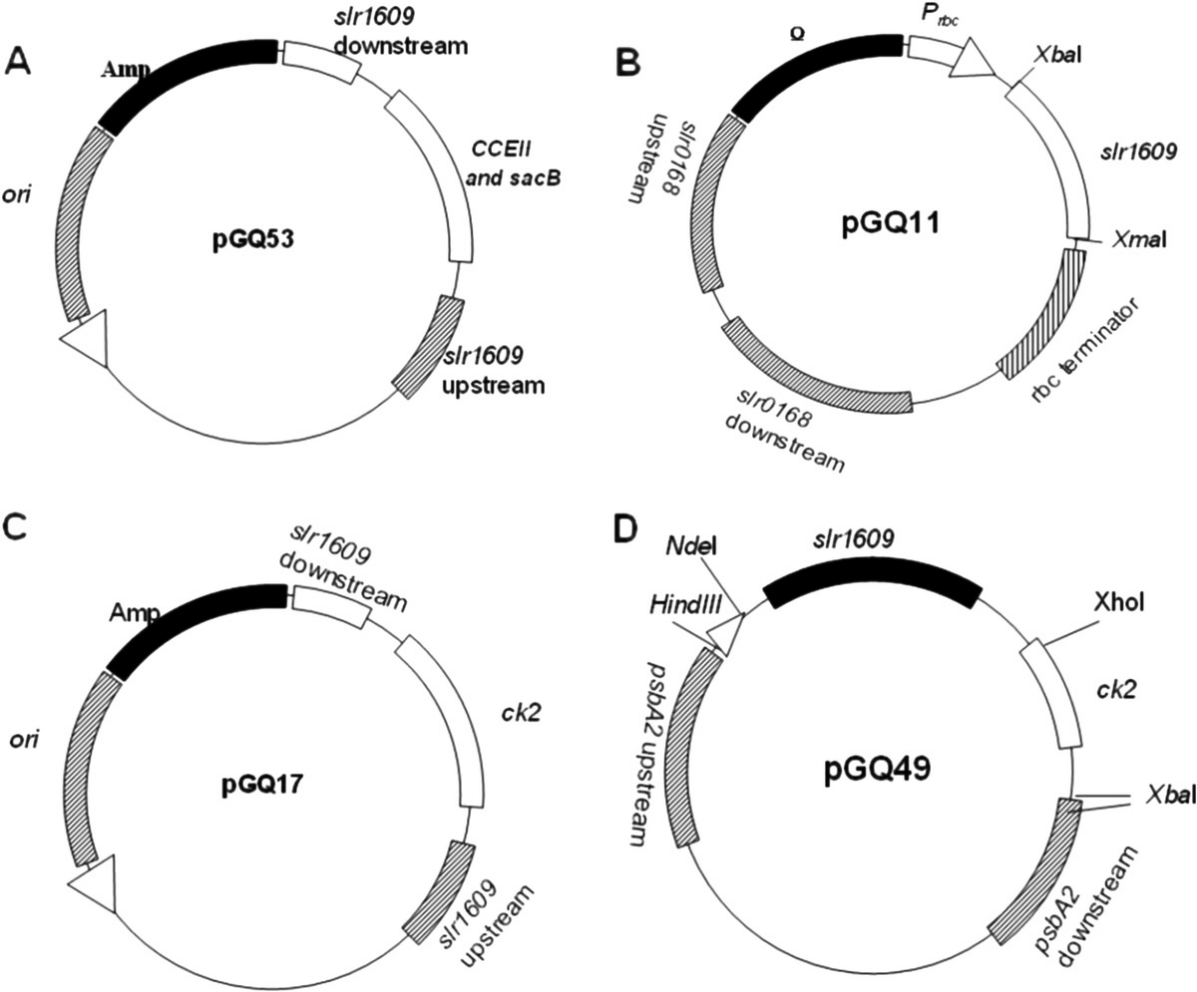
**Maps of the plasmids (A) pGQ53, (B) pGQ11, (C) pGQ17 and (D) pGQ49**.

To investigate the impact of AAS on production of free fatty acids and fatty acid derivatives, we constructed two plasmids pGQ11 (Figure 2B) and pGQ49 (Figure 2D) for over-expressing *slr1609 *gene, driven by a strong constitutive promoter *Prbc *or *PpsbA2 *and integrated into *slr0168 *[9] or *psbA2 *site, respectively. Two plasmids pGQ53 (Figure 2A) and pGQ17 (Figure 2C) were constructed for disruption of *slr1609 *gene with erythromycin or kanamycin resistance cassettes, respectively. The plasmid pGQ11 or pGQ53 was transformed into *Synechocystis *sp. PCC6803 generating GQ3 and GQ8 strains respectively for analysis of fatty acid and alkane production. The plasmid pGQ49 or pGQ17 was transformed into fatty-alcohol-producing strain Syn-XT14 generating GQ5 and GQ6 respectively for analysis of fatty alcohol production. Overexpressed AAS protein with C-terminal His-tag in GQ3 and GQ5 mutant were detected by western blotting as shown in Additional file 1: Figure 1.

**Figure 3:**
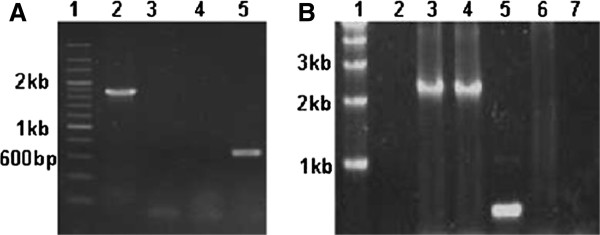
**PCR analysis of the genotype of *Synechocystis *sp. PCC 6803 mutant strains**. **(A) lane 1: **DNA marker (200 bp DNA Ladder Marker), **lane 2: **genomic DNA of GQ8 was amplified by primers kus and kdas outside the inserted gene segment, **lane 3: **plasmid pGQ53 was amplified by primers kvF and kvR located inside *slr1609 *gene (control), **lane 4: **genomic DNA of GQ8 was amplified by the same primers as lane 3, **lane 5: **genomic DNA of wild-type was amplified by the same primers as lane 3 (control). **(B) lane1: **DNA marker (1 kb DNA Ladder Marker), **lane2: **genomic DNA of wild type was amplified by primers 0168-2 and 1609NdeI (control), **lane3: **genomic DNA of GQ3 was amplified by the same primers as lane2, **lane4: **plasmid pGQ11 was amplified by the same primer as lane2 (control), **lane5: **genomic DNA of wild-type was amplified by primers 0168-1 and 0168-2(control), **lane6**: genomic DNA of GQ3 was amplified by the same primers as lane5, **lane7**: H_2_O was used as template, and the same primer as lane5 were used in PCR reaction(control).

**Figure 4:**
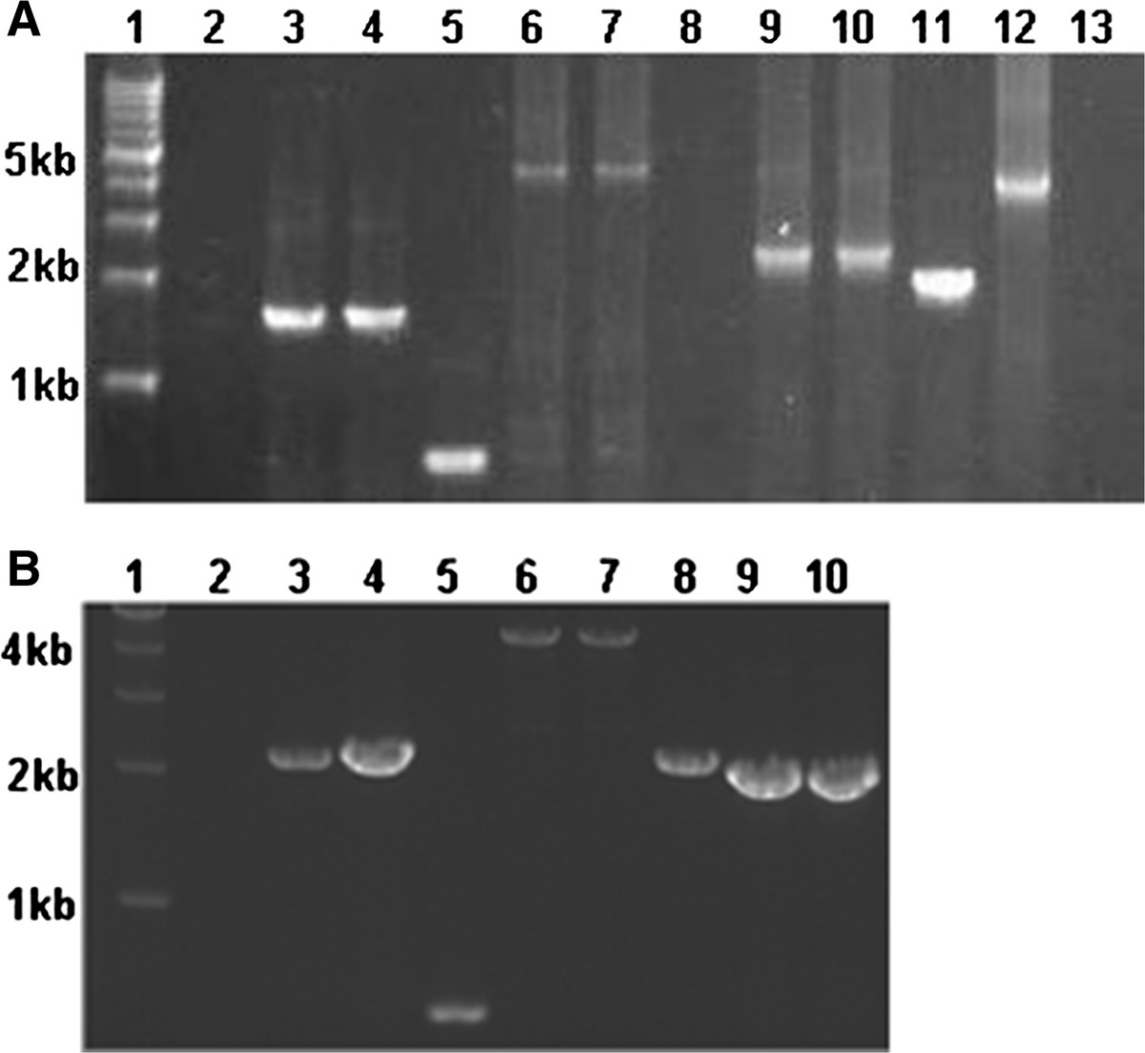
**PCR analysis of the genotype of *Synechocystis *sp. PCC 6803 mutant strains**. **(A) lane1: **DNA marker (1 kb DNA Ladder Marker), **lane2: **genomic DNA of wild-type was amplified by primers 0168-1 and far-1 (control), **lane3: **genomic DNA of GQ5 was amplified by the same primers as lane2, **lane4: **plasmid pXT14 was amplified by the same primers as lane2, **lane5: **genomic of wild-type was amplified by primers 0168-1 and 0168-2, **lane6: **genomic DNA of GQ5 was amplified by the same primers as lane5, **lane7: **plasmid pXT14 was amplified by the same primer as lane5 (control), **lane8: **genomic DNA of wild-type was amplified by primers pD1-3 and 1609R (control), **lane9: **genomic DNA of GQ5 was amplified by the same primers as lane8, **lane10: **plasmid pGQ49 was amplified by the same primers as lane8 (control), **lane11: **genomic DNA of wild-type was amplified by primers pD1-3 and pD1-2d-2 (control), **lane12: **genomic DNA of GQ5 was amplified by the same primers as lane11, **lane13: **plasmid pGQ49 was amplified by the same primers as lane11 (control). **(B) lane1: **DNA marker (1 kb DNA Ladder Marker), **lane2: **genomic DNA of wild-type was amplified by the primers 0168-1 and far-1 (control), **lane3: **genomic DNA of GQ6 was amplified by the same primers as lane2, **lane4: **plasmid pXT14 was amplified by the same primers as lane2 (control), **lane5: **genomic DNA of wild-type was amplified by primers 0168-1 and 0168-2 (control), **lane6: **genomic DNA of GQ6 was amplified by the same primers as lane5, **lane7: **plasmid pXT14 was amplified by the same primer as lane5 (control), **lane8: **genomic DNA of wild-type was amplified by primer 1609NdeI and 1609R (control, the size of target DNA fragment should be 2091 bp), **lane9: **genomic DNA of GQ6 was amplified by the same primers as lane8 (the size of target DNA fragment should be 1810 bp), **lane10: **plasmid pGQ17 was amplified by the same primers as lane8 (control, the size of target DNA fragment should be 1810 bp).

Due to cyanobacterial cells containing multiple copies of chromosomes [10], the complete replacement of wild type alleles must be established and confirmed by PCR (Figure 3 and 4). Primers used in this study were listed in Additional file 1: Table 1. Because the whole inserted fragment is too long to amplify from genomic DNA, the over-expressed genes were checked by two reactions for successful insertion and correct orientation of the *slr1609 *or FAR gene and complete replacement of wild-type alleles (Figure 3B and 4). The first reaction with the primer (0168-2 or Pd1-3) of insert site and primer (1609NdeI, 1609R or far-1) for the inserted gene verified the genes were inserted in the correct orientation. The second reaction with primers (0168-1/0168-2 or pD1-1/p pD1-2d-2) of inserted site verified the wild-type was replaced completely (Figure 3B and 4). The disrupted genes were checked with internal primers (Figure 3A and 4B) or primers that flanked the insertion site to prove the wild-type allele was replaced completely. The results of the PCR indicated the correct mutants were constructed.

### The amount of free fatty acids can be doubled in the *Synechocystis *mutant strain with *slr1609 *knockout

**Figure 5:**
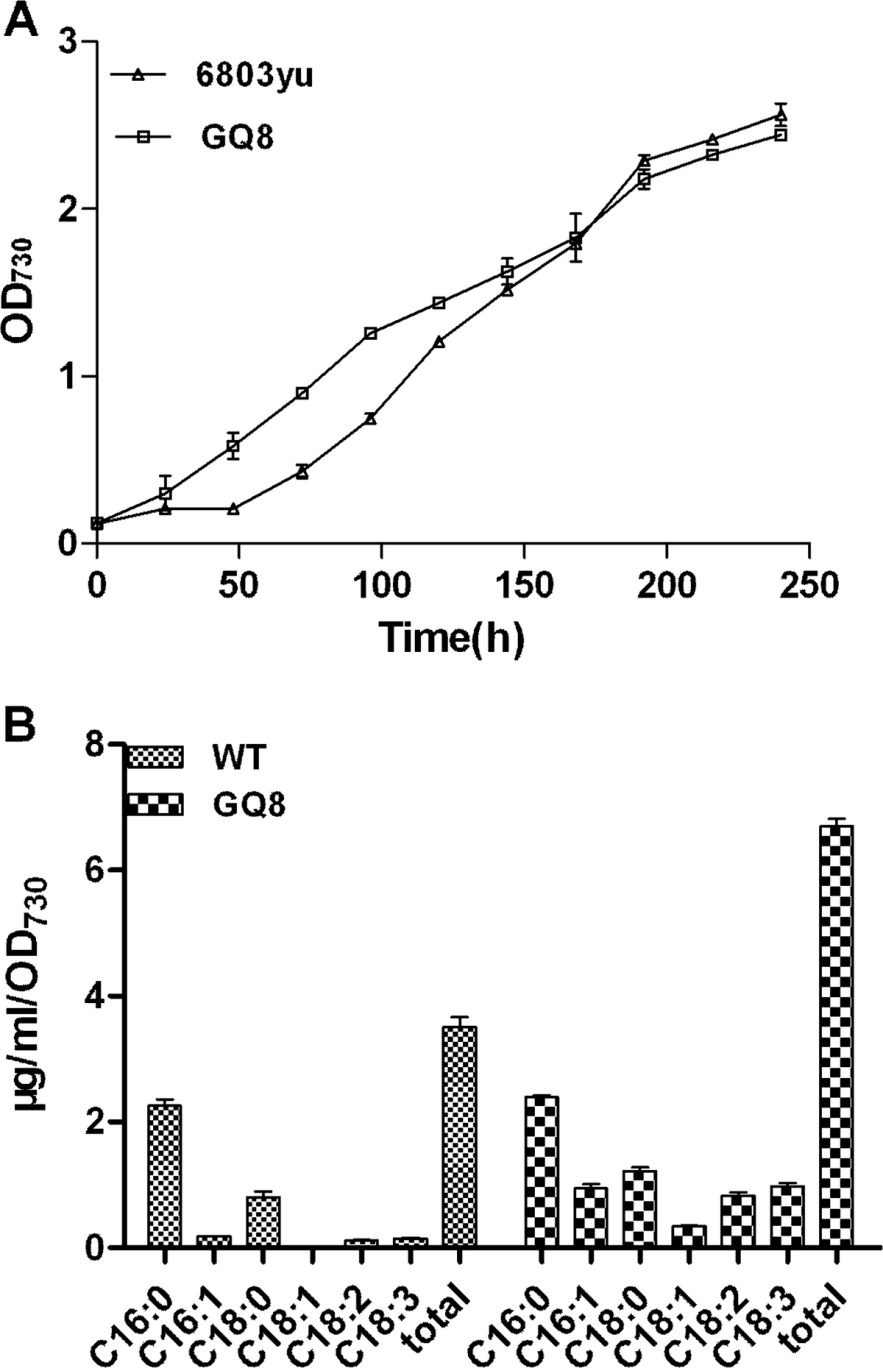
**Comparison of cell growth (A) and production of free fatty acids (B) between *Synechocystis *sp. PCC 6803 wild-type strain labeled as 6803yu and the mutant strain GQ8 with the deletion of *slr1609 *gene**.

The total free fatty acids of the wild-type strain and the mutant strain GQ8 with *slr1609 *deletion were extracted using the methods described in the Experimental Procedures. The wild-type and the mutant strain displayed similar growth behaviors (Figure 5A). However, the content of free fatty acids showed substantial differences between two strains (Figure 5B). In the *slr1609 *deletion mutant, the concentration of total free fatty acids was 6.7 ± 0.2 μg/mL/OD, while that of the wild type was 3.5 ± 0.25 μg/mL/OD. The deletion of *slr1609 *increased free fatty acid accumulation close to two folds. It indicates that the dysfunction of fatty acid activation caused by the deletion of *slr1609 *results in an increase of free fatty acid accumulation.

As to the contents of the pool of free fatty acids, the amount of unsaturated fatty acids with carbon chain length of C16 and C18 was significantly higher in the *slr1609 *knockout mutant strain compared to the wild-type strain. Double bonds can only be introduced into free fatty acid coupled to the glycerol backbone of membrane lipids by acyl-lipid-type desaturases. Indicating that unsaturated free fatty acids being released from membrane lipids of senescent or damaged cells, while unsaturated free fatty acids in AAS deletion mutant can not be recycled and incorporated to membrane lipids.

In the mutant strain GQ3 with *slr1609 *over-expression, there is no significant change to the production of free fatty acids compared to the wild-type strain (data not shown). It has been confirmed that free fatty acids are released from membrane lipids in *Synechocystis *sp. PCC6803 [10]. Indicating free fatty acid production is not only determined by the fatty acyl-ACP pool size, but also by the biosynthesis of membranes and hydrolysis of membrane lipids which are physiologically regulated.

### The production of alkanes was significantly reduced in the *slr1609 *deletion mutant strain

**Figure 6:**
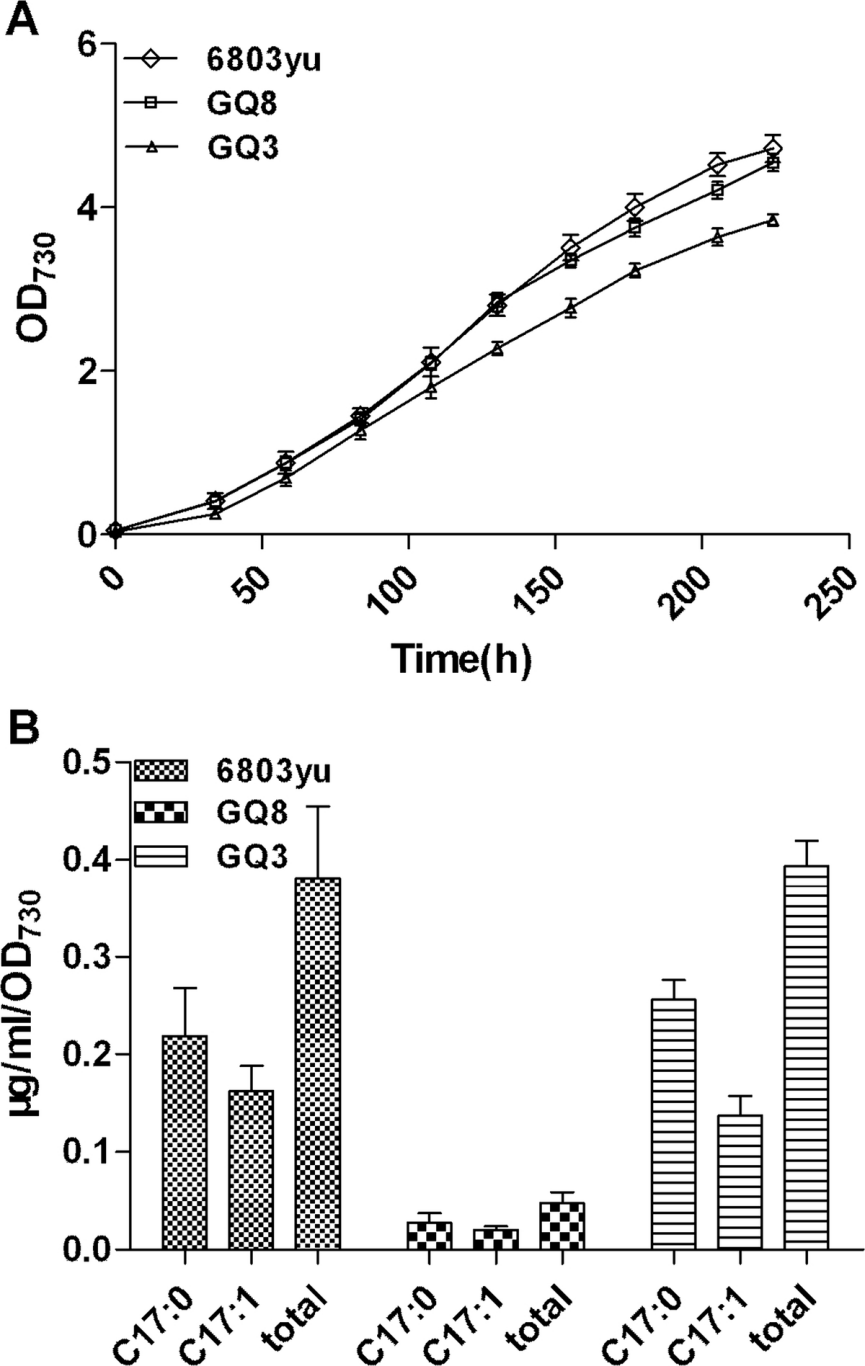
**Comparison of cell growth (A) and production of fatty alkanes (B) in *Synechocystis *sp. PCC 6803 wild-type strain labeled as 6803yu, the mutant strain GQ8 with the deletion of *slr1609 *gene and GQ3 with the overexpression of *slr1609 *gene**.

Alkanes are the predominant constituents of gasoline, diesel, and jet fuels. Production of alkanes has been reported in a diversity of cyanobacteria, with heptadecane and heptadecene being the most abundant hydrocarbons found in *Synechocystis *sp. PCC6803. In this pathway fatty acyl-ACP is reduced to a fatty aldehyde by a fatty acyl-ACP reductase (AAR) and then the fatty aldehyde decarbonylase (ADC) is able to convert the aldehyde into an alkane. Besides the fatty acyl-ACP produced by *de novo *fatty acid synthesis from acetyl-CoA, acyl-ACP synthetase (AAS) is essential for recycling fatty acids into fatty acyl-ACP. The results showed that the production of hydrocarbons was significantly reduced by around 90% in the mutant strain GQ8 with an *slr1609 *deletion (0.047 ± 0.01 μg/mL/OD) compared with the wild-type strain (0.38 ± 0.07 μg/mL/OD) (Figure 6B), and this indicates that AAS plays an essential role in alkane biosynthesis. AAS can enhance the total amount of fatty acyl-ACP available for alkane production, and the acyl-ACP formed by AAS activity may be more accessible to the acyl-ACP formed by *de novo *fatty acid synthesis.

The production of alkanes was not enhanced by the over-expressing *slr1609 *gene alone in the GQ3 strain (0.39 ± 0.03 μg/mL/OD)(Figure 6B). Due to the activities of downstream enzymes of the alkane producing pathway, AAR and ADC, are rather low and fatty acyl-ACPs might not be efficiently converted to alkanes [11]. Fatty acyl-ACPs are also a supplier of fatty acyl groups for biosynthesis of lipid A [12], phospholipids [13], and membrane-derived lipo-polysaccharides [14].

### *Synechocystis *AAS plays an important role in fatty alcohol production

**Figure 7:**
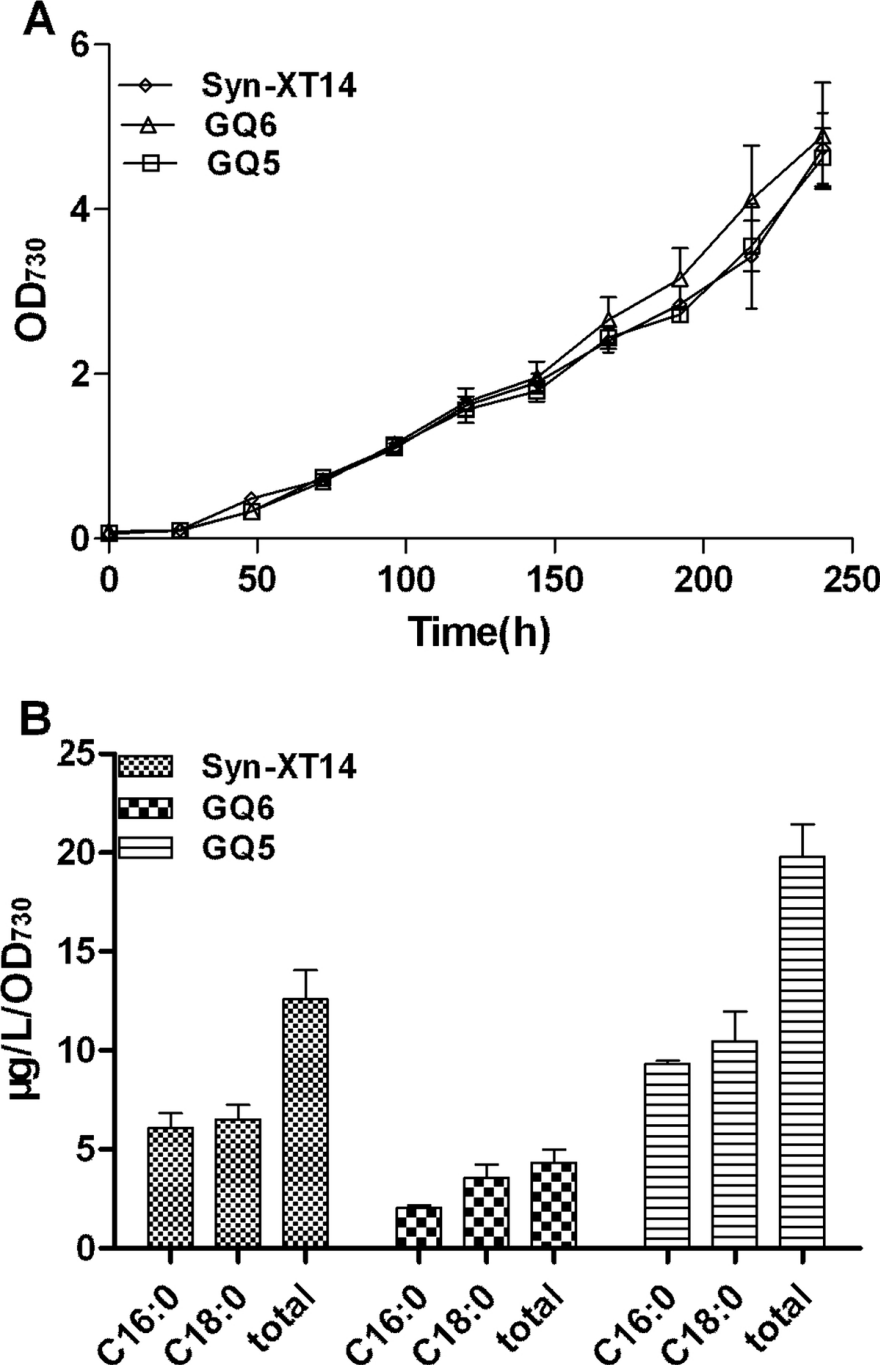
**Comparison of cell growth (A) and production of fatty alcohols (B) in engineered fatty alcohol-producing strain Syn-XT14, GQ5 with the overexpression of *slr1609 *gene and GQ6 with the deletion of *slr1609 *gene**.

Fatty alcohols possess carbon chain length which range from C8 to C22, and can be used as detergents, precursors for synthesis of other chemicals or fuels. We have constructed a fatty-alcohol-producing strain Syn-XT14, by the introduction of a jojoba acyl-CoA reductase gene into wide-type strain in previous work [4], and the effect of *Synechocystis *AAS on fatty alcohol production were examined by over-expressing (GQ5) or deleting the *slr1609 *gene (GQ6) in Syn-XT14. The results showed that fatty alcohol production was enhanced by about 60% in GQ5 (19.8 ± 2.3 μg/L/OD) or decreased by about 60% in GQ6 (4.9 ± 0.1 μg/L/OD) compared with Syn-XT14 (12.5 ± 2.0 μg/L/OD), respectively (Figure 7). The data indicates that *Synechocystis *AAS plays an important role in fatty alcohol production.

Although the native jojoba FAR has a preference for very-long-chain acyl-CoA substrate (C20, C22 and C24), assays of jojoba extracts indicated that it is capable of reducing C16:0-ACP and C18:0-ACP [7]. It's a reductase with broad substrate specificity. It may be possible that the acyl-ACP produced by AAS can also be accepted as substrate in addition to acyl-CoA by jojoba FAR in engineered *Synechocystis *strains. It is also possible that the acyl-ACPs, which are synthesized by *Synechocystis *AAS, could be in turn transacylated to acyl-CoAs by a reverse catalysis of acetyl-CoA-ACP-transacylase (EC 2.3.1.38) type reaction.

## Conclusions

In this study the effects of fatty acid activation functionalized by a fatty acyl-ACP synthase on the production of fatty acid-based biofuels including fatty alcohols and alkanes in a photosynthetic cyanobacterium were evaluated and analyzed. We found fatty acid activation to be essential for efficient production of alkanes and plays a key role in manipulating fatty alcohol production. The results here provide promising clues for metabolically engineering cyanobacteria to improve photosynthetic production of fatty acid-based biofuels.

## Methods

### Chemicals and reagents

Pentadecanol, eicosane and nonadecanoic acid were obtained from Sigma-Aldrich (USA). Other chemicals were from Merck (Germany) or Ameresco (USA). Oligo nucleotides and gene synthesis were carried out by Sangon (Shanghai, China). Taq DNA polymerases and all restriction endonucleases were from Fermentas (Canada) or Takara (Japan). The DNA ladders were from Takara (Japan). The kits used for molecular cloning were from Omega (USA) or Takara (Japan).

### Construction of *Synechocystis *sp. PCC683 mutant strains

All primers used in this study are listed in the Additional file 1.

The *slr1609 *gene was amplified from the genomic DNA of *Synechocystis *sp. PCC6803 with the primers 1609NdeI/1609R and subcloned into *Nde*I*/Xho*I site of the plasmid pET21b (Novagen, USA) to generate the plasmid pGQ7. The gene was cloned from pGQ7 with the primers 1609XbaI/1609DraI and subcloned into *Xba*I/*Sma*I site of the plasmid pFQ20 [4] to generate the plasmid pGQ11.

The plasmid pXT68 was constructed based on the site of *psb*A2 gene. Both upstream and downstream fragments of *psb*A2 gene were cloned from the genomic DNA of *Synechocystis *sp PCC6803 with the primers Pd1-2-f/Pd1-2-r and pD1-2 d-1/pD1-2 d-2 respectively and inserted into the TA cloning site of pMD18-T, to generate the plasmids pXT25 and pXT59. The kanamycin resistance (kan^r^) gene (*ck2*) cassette was excised with *Eco*RV and *Xba*I from pRL446 [15] and inserted into the *Pst*I site of pXT25 with blunt ends, to generate the plasmid pXT62. The 4.5 kb fragment containing *ck*2 and upstream of *psb*A2 was excised with *Xba*I and *Sph*I from pXT62 and inserted into *Xba*I site of pXT59 with blunt ends, to generate the plasmid pXT68. Then, the *slr1609 *gene was excised with *Nde*I and *DraIII* (blunted end) from pGQ7 and inserted into the *Nde*I/*Sal*I (blunted end) site of pXT68, to generate the plasmid pGQ49.

The plasmids pGQ17 and pGQ53 were constructed and used to disrupt the *slr1609 *gene via homologous recombination in *Synechocystis *[16]. Genomic DNA was used as the template to amplify the 500 bp N-terminal and C-terminal fragments of *slr1609 *ORF using the primers 1609 kuF/R and 1609 kdF/R, respectively. The N-terminal and C-terminal fragments were cloned into pMD18-T to generate the plasmids pGQ12 and pGQ13, respectively. The *ck2 *gene from pRL446 [15] was cloned into the *Bam*HI site of pGQ12 to generate plasmid pGQ14. After digestion with *Dra*I and *Eco*RI and blunting by T4 DNA polymerase, the *ck2 *gene together with N-terminal fragment of *slr1609 *from pGQ14 were cloned into the *Sma*I site of plasmid pGQ13 to generate plasmid pGQ17. The erythromycin resistance gene *cce2 *was digested with *Eco*RV from pRL271 [17] and cloned into the blunted *Bam*HI site of pGQ17 to generate plasmid pGQ53. Plasmids constructed and used in this study were listed in Table 1.

**Table 1:** Plasmids constructed and used in this study

Plasmid	Relevant characteristics^a, b^	Reference
pGQ7	Ap^r^, pET21b derivative containing *slr1609gene*, T7 promoter	This study

pXT25	Ap^r^, pMD18-T derivative containing upstream fragment of *psbA2*, T7 promoter	This study

pXT59	Ap^r^, pMD18-T derivative containing downstream fragment of *psbA2*, T7 promoter	This study

pXT62	Ap^r^, Kan^r^, pMD18-T derivative containing upstream fragment of psbA2 and *CK2*	This study

pXT68	Ap^r^, Kan^r^, pMD18-T derivative containing upstream and downstream fragments of *psbA2, CK2 *and *sacB*	This study

pFQ20	Ap^r ^Spe^r^, pKW1188SL derivative containing *lacZ*, P_rbc _promoter	[4]

pGQ11	Ap^r ^Spe^r ^, pFQ20 derivative containing *slr1609*, P_rbc _promoter	This study

pGQ49	Kan^r^, pXT68 derivative containing *slr1609*, P_psbA2 _promoter	This study

pGQ12	Ap^r^, pMD18-T derivative containing upstream fragment of *slr1609*, T7 promoter	This study

pGQ13	Ap^r^, pMD18-T derivative containing downstream fragment of *slr1609*, T7 promoter	This study

pGQ14	Ap^r^, Kan^r^, pMD18-T derivative containing upstream fragment of *slr1609 *and *CK2*	This study

pGQ17	Ap^r^, Kan^r^, pMD18-T derivative containing upstream and downstream fragments of *slr1609 *and *CK2*	This study

pGQ53	Ap^r^, Cm^r^, Em^r^, pMD18-T derivative containing upstream and downstream fragments of *slr1609, CCEII *and *sacB*	This study

pXT14	Spe^r^, pFQ20 derivative containing FAR gene (jojoba), P_rbc _promoter	[4]

^a ^Ap, Ampicillin. Spe, Spectinomycin. Kan, kanamycin. Cm, Chloramphenicol. Em, Erythromycin.

^b^*CK2 *gene for kanamycin antibiotic resistance and *CCEII *gene for erythromycin antibiotic resistance.

All of the constructs were checked by enzyme digestion and then transformed to *Synechocystis *sp. PCC6803 cells [18]. The plasmids pGQ11 and pGQ53 were transformed to *Synechocystis *sp. PCC6803 wild-type to generate the mutant strains GQ3 and GQ8 respectively. The plasmids pGQ17 and pGQ49 were transformed to *Synechocystis *sp. PCC6803 mutant strain Syn-XT14 [4] to generate two new mutant strains GQ6 and GQ5 respectively. For the initial selection of transformants, the DNA/cell mixture was applied to BG11 agar plates. After 18 h the membrane filters were applied to fresh BG11 agar plates containing antibiotics (10 μg mL^-1 ^spectinomycin, 10 μg mL^-1 ^erythromycin or 5 μg mL^-1^/5 μg mL^-1 ^spectinomycin/kanamycin). Homogeneous mutants were obtained by successive streaking on BG11 plates containing antibiotics. Complete segregation of all mutants was verified by employing PCR. Strains constructed and used in this study were listed in Table 2.

**Table 2:** Strains constructed and used in this study

Strain	Genotype^a,^	Reference
6803yu	*Synechocystis *sp. PCC6803 Wild-type, Glucose-tolerance	Prof. Xu X.

GQ3	*slr0168*∷Omega *P*_rbc_*slr1609*	This study

GQ8	*slr1609*∷*CCEII*	This study

GQ5	*slr0168*∷omega *P*_rbc _*far *(jojoba), *psbA2*∷*CK2 P*_psbA2_*slr1609*	This study

GQ6	*slr0168*∷ omega *P*_rbc _*far *(jojoba); *slr1609 *∷*CK2*	This study

Syn-XT14	*slr0168*::Omega *P*_rbc _*far *(jojoba) *T*_rbc_	[4]

*^a ^P*_petE_, 0.4 kb DNA fragment containing the promoter of *petE *gene. *P*_rbc_, 0.3 kb DNA fragment containing the promoter of *rbc *operon. *T*_rbc_, 0.2 kb DNA fragment downstream of *rbcS *gene. *P*_psbA2_, 1.5 kb DNA fragment containing the promoter of *psbA2 *gene. All promoters and terminators mentioned here are from *Synechocystis *sp. PCC 6803.

### Cultivation of *Synechocystis *sp. PCC683 strains

Liquid cultures of *Synechocystis sp*. PCC 6803 were grown photo-autotrophically in BG 11 media [19] at 30°C under constant illumination at a photosynthetic photon flux density of approximately 30 μmol photons m^-2 ^s^-1 ^and with aeration by sterile air or in a shaker. When necessary, the following antibiotics were added: erythromycin (20 μg mL^-1^) and spectinomycin (20 μg mL^-1^)_. _Growth was monitored by following the OD at 730 nm. The *Synechocystis *sp. PCC6803 wild-type strain, the mutant strains GQ8 with deletion of the *slr1609 *gene and GQ3 with overexpression of the *slr1609 *gene were respectively grown in 100 mL Erlenmeyer flask containing 50 mL of BG11 medium in a shaker for free fatty acid analysis. The *Synechocystis *sp. PCC6803 wild-type strain, the mutant strains Syn-XT14 with overexpression of the FAR gene, GQ6 with deletion of the *slr1609 *gene and GQ5 with overexpression of the *slr1609 *gene were respectively grown in a 500 mL Erlenmeyer flask containing 300 mL of BG11 medium with aeration by sterile air for fatty alkane or fatty alcohol analysis. The *Synechocystis *sp. PCC6803 wild-type strain, the mutant strains GQ8 with deletion of the *slr1609 *gene and GQ3 with overexpression of the *slr1609 *gene were respectively grown in a 500 mL Erlenmeyer flask containing 300 mL of BG11 medium with aeration by sterile air for fatty alkane analysis.

### Extraction and analysis of free fatty acids, fatty alkanes and fatty alcohols

For extraction of free fatty acids, 20 mL of the culture was lysed by sonication (total 30 min with 10 s on and 5 s off intervals) when the stationary phase (about 240 h) reached. To each 20 mL aliquot, 20 mL of 2:1 (v/v) CHCl_3_:CH_3_OH were added and the resulting mixture was mixed well [20]. For GC-MS analysis of free fatty acids, 10 μg of nonadecanoic acid was added as the internal standard. A two-phase system (top: aqueous, bottom: organic) was generated after shaking for 1 h and centrifugation at 3000 rpm at room temperature for 5 min. The bottom organic phase was collected and concentrated under a stream of nitrogen at 55°C giving a residue that was resuspended in 600 μL of hexane. Aliquots of this mixture were analyzed by using GC-MS with an Agilent 7890A-5975 C system equipped with Agilent HP-INNOWax (30 m × 250 μm × 0.25 μm). Helium (constant flow 1 mL/min) was used as the carrier gas. The temperature of the injector was 250°C and the following temperature program was applied: 100°C for 1 min, increase of 5°C min^-1 ^to 200°C then increase of 25°C min^-1 ^to 240°C for 15 min.

Previous work in our lab showed that fatty alcohol and alkane can not be detected in relative culture media (data not shown). For extraction of fatty alkanes, *Synechocystis *cells at stationary phase (about 240 h) were harvested from 200 mL of culture by centrifugation. The cells were resuspended in 10 mL of TE buffer (pH8.0) and then lysed by sonication. The lysate added with 30 μg of eciosane as internal standard was extracted for 1 h at room temperature with 10 mL of 2:1 (v/v) CHCl_3_:CH_3_OH. The same following sample preparation and GC-MS analysis methods described above were used for fatty alkane analysis.

The same extraction methods described above for fatty alkane analysis were used for fatty alcohol, except adding 20 μg of 1-pentadecanol as the internal standard. The following temperature program was applied here: 50°C for 1 min, increase of 20°C min^-1 ^to 180°C then increase of 10°C min^-1 ^to 240°C for 20 min.

## Abbreviations

ACP: acyl carrier protein; AAR: acyl-ACP reductase; ADC: aldehyde decarbonylase; FAR: fatty acyl-CoA reductase; ACS: acyl-CoA synthetase; AAS: acyl-ACP synthetase; ACC: acetyl-CoA carboxylase; FAS: fatty acid synthase; GPAT: glycerol-3-phosphate-acyl transferase; ACAT: acyl-CoA-ACP transferase; ACR: acyl-CoA reductase.

## Competing interests

The authors declare that they have no competing interests.

## Authors' contributions

XL conceived of the study. XL and QG designed the experiments. QG carried out the construction and cultivation of *Synechocystis *sp. PCC683 mutant strains. QG carried out extraction and analysis of free fatty acids, fatty alkanes and fatty alcohols. WW and HZ participated in GC-MS analysis. XL, QG and WW wrote the manuscript. All authors read and approved the final manuscript.

## Supplementary Material

Additional file 1 **Figure **1. Western blot analysis of overexpressed AAS protein in GQ3 and GQ5 mutant with anti-His-tag antibody. **Table **1. Primers used in this study. Click here for file
