# Supplementary material for: Effects of fatty acid activation on photosynthetic production of fatty acid-based biofuels in Synechocystis sp. PCC6803
Source: Biotechnol Biofuels. 2012 Mar 21;5:17. doi: 10.1186/1754-6834-5-17 (PMC3366867; doi:10.1186/1754-6834-5-17)
Supplement: Additional file 1 — Figure 1. Western blot analysis of overexpressed AAS protein in GQ3 and GQ5 mutant with anti-His-tag antibody. Table 1. Primers used in this study. [file 1754-6834-5-17-S1.PDF]

Additional file

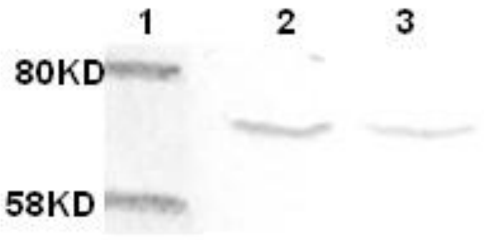

Figure. 1. Western blot analysis of overexpressed AAS protein in GQ3 and GQ5 mutant with anti-His-tag antibody. Lane 1: protein marker, Lane 2: soluble extracts from samples of GQ3, lane 3: soluble extracts from samples of GQ5.

Table 1 Primers used in this study.

primer	sequence	reference
1609NdeI	5'GGCATATGGAAAAGAG ATATTCTAACGTAGTAGCA3'	This study
1609R	5'CCCTCGAGGGCAAACATGGCGTTG3'	This study
1609XbaI	5'GCTCTAGAATGGACAGTGGCCATG3'	This study
1609DraI	5'TTCTTTAAATCAGTGGTGGTGGTGG3'	This study
1609kuF	5'TTTAAATGGTGATGAACACTGGGGA3'	This study
1609kuR	5'GGGATGACTATGGCGATCGTTGAG3'	This study
1609kdF	5'TGTTTACGCAGTGCCTACATTGA3'	This study
1609kdR	5'CCCATAGGCCTTAGATCGTGTTT3'	This study
Pd1-2-f	5'CACATAGATCTGCCAGTTGAGGT3'	This study
Pd1-2-r	5'GGGCATATGGTTATAATTCCTTATGTATTTG3'	This study
kvF	5'TTTAAATGGTGATGAACACTGGGGA3'	This study
kvR	5'CCCATAGGCCTTAGATCGTGTTT3'	This study
0168-1	5'ACCTCTCCACGCTGAATTAG3'	[4]
0168-2	5'TTCCAGGCCACATTGTTGTC3'	[4]
pD1-2d-1	5'TTCCTTGGTGTAATGCCA ACTG3'	This study
pD1-2d-2	5'TCCACACTGGGAAGTTTGCC3'	This study
pD1-3	5'AGCTTCGTGTATATTAACTTCCTGT3'	This study
Far-1	5'GGGTCTAGAATGGAAGAGATGGGCAGCATC3'	[4]
